# Contraception: Influence on Vaginal Microbiota and Identification of Vaginal Lactobacilli Using MALDI-TOF MS and 16S rDNA Sequencing

**DOI:** 10.2174/1874285801812010218

**Published:** 2018-06-29

**Authors:** Sonia E. Fosch, Cecilia A. Ficoseco, Antonella Marchesi, Silvina Cocucci, Maria E. F. Nader-Macias, Beatriz E. Perazzi

**Affiliations:** 1 Agencia PROSAR Santa Fe Norte, Fundación Bioquímica Argentina, Santa Fe, Argentina; 2 Servicio de Atención Médica, Ministerio de Salud, Sa Pereira, Argentina; 3 CERELA-CONICET (Centro de Referencia para Lactobacilos-Consejo Nacional de Investigaciones Científicas y Técnicas), San Miguel de Tucumán, Argentina; 4 Laboratorio de Bacteriología Clínica, Departamento de Bioquímica Clínica. Hospital de Clínicas, Facultad de Farmacia y Bioquímica, Universidad de Buenos Aires, Buenos Aires, Argentina.

**Keywords:** Contraception, Basic vaginal states, Vaginal microbiota, Lactobacilli, Probiotic, Immune activation

## Abstract

**Background:**

The vaginal microbiome is influenced by a wide variety of factors, including contraceptive methods.

**Aim:**

To evaluate the effect of contraceptive methods on vaginal microbiota and to compare MALDI-TOF MS and 16S rDNA sequencing for lactobacilli identification.

**Patients and Methods:**

One hundred and one (101) women consulting for birth control were included in a prospective study. Their vaginal content was sampled and analyzed once before they started using the contraceptive method of their choice, and twice after the initiation of contraception, at three months (94/101 women attended) and at six months (89/101 women attended). The relative frequencies of yeasts and trichomonas were analyzed. MALDI-TOF MS and 16S rDNA sequence analysis were applied for the identification of lactobacilli in their vaginal microbiota. The following contraceptive methods were assessed: Combined Oral Contraceptive Pill (COCP), Condom (CON) and The Rhythm Method (RHYT). McNemar’s statistical test was applied.

**Results:**

A statistically significant association between COCP and normal microbiota was observed after three months (*p*< 0.01) and after six months (*p*< 0.0001), when the vaginal microbiota was modified. At six months, inflammatory reaction was detected in 3/7 women in the CON group, while 6/7 patients using RHYT showed the same state. Yeast colonization increased with the COCP. Identification of lactobacilli by MALDI-TOF MS analysis compared to 16S rDNA sequencing yielded 92.9% concordant results. *Lactobacillus gasseri* and *L. crispatus* were the predominant species.

**Conclusion:**

The pattern of vaginal states was significantly modified. Hormone administration apparently corrected the alterations and retained a normal vaginal state. MALDI-TOF MS has the potential of being an accurate tool for the identification of vaginal lactobacilli species *L. murinus* was for the first time isolated from the vagina.

## 1 INTRODUCTION

The vaginal microbiota provides the first line of defense for the lower genital tract. Despite its importance, it is still necessary to learn more about these microbial communities that vary among individuals or in their composition and function, but more importantly, about how their constituent members interact with one another to form a dynamic ecosystem that responds to external factors.

The microenvironment, with its vaginal microbiota primarily regulated by a hormonal immune system, can be protected or altered by a wide variety of factors, including contraceptive methods. Some studies reported that combined oral contraceptives are associated with the stability of the vaginal microbiota, while the use of male condom is associated with Vaginal Inflammatory Reaction (VIR). However, the rhythm method (*i.e.*, sexual abstinence from days 10 to 18) did not produce any significant changes [1-4].

Culture-dependent techniques and microscopic observation of the “normal” Vaginal Content (VC) typically show a predominance of *Lactobacillus* species, which are believed to promote a healthy vaginal milieu by providing numerical dominance, but also by producing lactic acid to maintain an acid environment, thus inhibiting different types of pathogenic or potentially pathogenic microorganisms, showing a negative correlation with Bacterial Vaginosis (BV) [5]. Lactobacilli also produce hydrogen peroxide [6], organic acids and bacteriocins [7], and are included in probiotic products considered to be beneficial to human health [8]. It has been demonstrated that only some specific *Lactobacillus* strains express beneficial properties, with differences among species and even among strains within the same species [9].

Molecular-based techniques have shown that a healthy vaginal microbiota does not contain a wide variety of *Lactobacillus* species. One or two lactobacilli from three to four species (mainly *L. crispatus*, *L. jensenii* and *L. gasseri*, and in some cases *L. iners*) are dominant, whereas other species are rare, fewer, and apparently belong to novel phylotypes [10-12]. Ravel *et al.* have published a study in which the production of lactic acid may be conserved in communities dominated by lactobacilli, and also in communities with low proportion of lactic acid bacteria and high proportion of strictly anaerobic organisms [13].

Moreover, there are a number of theories that attempt to explain the ecological dynamics of vaginal ecosystems since they respond to some disturbances caused by human activities such as intercourse, contraceptive practices and other habits [1-4, 14]. This knowledge is required to diagnose and assess the dysbioses of the vaginal tract given their probable association with inflammatory or immune activation cascades and adverse clinical outcomes like preterm birth and miscarriage, pelvic inflammatory disease, and the acquisition and transmission of sexually transmitted infections, including HIV [15].

The aims of this study were to evaluate the effect of contraceptive methods on vaginal microbiota and to compare MALDI-TOF MS and 16S rDNA sequencing for lactobacilli identification.

## 2 MATERIALS AND METHODS

This substudy included 101 of 215 women who attended for birth control at a public health care center in Sa Pereira (Santa Fe, Argentina) from 2013 to 2015. These 101 women were selected at random to evaluate the influence of contraception in two time points, at three months (94/101 women attended) and at six months (89/101 women attended) and to incorporate the identification of cultivable vaginal *Lactobacillus* species as a continuation of the research started in 2013 [3].

The target population ranged in age from 14 to 45 (mean 28.7 years old), was sexually active—from an average of one to two heterosexual relationships per week—and had a high degree of social homogeneity.

The study was approved by the Ethics Committee of Hospital de Clínicas on July 5, 2013, Buenos Aires, Argentina. All the women participating in this study signed an informed consent.

Vaginal discharge samples were extracted from the vaginal fornix with swabs at three different times: when the women began using the contraceptive method of choice, and three and six months later. Samples were obtained between days 10 and 20 of the sexual cycle.

Exclusion criteria were: Women undergoing local or systemic treatment with antibiotics, antifungals, corticosteroids, antiparasitic drugs or immunosuppressants, and women with some degree of mental disability.

The study was prospective, consecutive, experimental and descriptive. Vaginal contents were evaluated when the women began using the method of choice and their BVSs were established by applying the Balance of Vaginal Content (BAVACO) methodology [16]. The evolution of their BVSs was analyzed according to the type of contraceptive method used.

The BAVACO methodology includes the morphological analysis of the vaginal sample, based on the relationship between the Numerical Value (NV) using Nugent method and the VIR. The numerical value, which scoring systemis 0 to 10, it was obtained as a weighted combination of the following morphotypes: Gram-positive bacilli compatible with lactobacilli, gram-variable cocobacilli compatible with Gardnerella and anaerobes and curved gram-variable bacilli. A VIR was considered positive if there were more than 5 leukocytes (reading in Giemsa, using 1000X). Five BVSs were defined: normal microbiota (I), normal microbiota with inflammatory reaction (II), intermediate microbiota (III), bacterial vaginosis (IV) and nonspecific microbial vaginitis (V) (Table **1**). The frequency of each BVS was evaluated according to the contraceptive method used and the relative frequencies of yeasts and trichomonas associated with each BVS.

The women included in the study used the following contraceptive methods uninterruptedly for three and six months: combined oral contraceptive pill (levonorgestrel-ethinylestradiol) (COCP), condom (CON) and the rhythm method (RHYT) (*i.e.*, sexual abstinence between days 10 and 18 of the cycle).

The vaginal samples were inoculated into the following culture media (incubated in 5% CO_2_ atmosphere, for 48 hours):

- Columbia base agar (Britania, Argentina) enriched with 5% heme and De-Man, Rogosa and Sharpe agar (MRS, Biokar Diagnostics, France) to isolate lactobacilli.
- Modified thioglycolate to increase sensitivity for detection of *Trichomonas vaginalis* after a seven-day incubation period [17].
- Chromogenic medium for the isolation and presumptive differentiation of prevalent *Candida* species (CHROMagar Microbiology, France).

After the incubation conditions described, the isolated colonies of each medium, which were previously characterized by their morphology and basic phenotypic properties as gram-positive, catalase-negative bacilli, Strains were transferred to MRS plates for proteomic and rep-PCR fingerprinting.

All isolates from MRS agar were analyzed by MALDI-TOF MS (Bruker BD); using a reference database that included over 90 *Lactobacillus* strains [18]. The identification result was considered reliable when at least the two best matches (log-score ≥ 1.70) obtained with the MALDI Biotyper database indicated the same species.

Extraction of chromosomal DNA from lactic acid bacteria was carried out according to the modified Pospiech and Neumann technique [19]. The rep-PCR fingerprinting method was used for clustering the isolated strains using the (GTG) 5 primers [19]. Amplification reactions were performed in a Bio Rad My Cycler TM thermocycler. PCR products were separated electrophoretically on 1.5% agarose gel. Genomic DNA from selected strains in each cluster was used for amplification of the 16S rDNA gene using primers MLB and PLB to be later sequenced [20]. For species identification, the 16S rDNA sequences were compared considering a homology percentage higher than 98%. rRNA gene sequence alignments were performed using the multiple sequence alignment method and identification queries were fulfilled by a BLAST search in GenBank (http://www.ncbi.nlm.nih.gov/GenBank/).

### 2.1 Statistical Analyses

The evolution of the frequency of the BVSs in the COCP group was analyzed with McNemar’s test. A *p* value < 0.05 was considered statistically significant. The Kappa index was calculated as a measure of agreement between the sequencing of the 16S rDNA gene and MALDI-TOF MS for the identification of lactobacilli.

## 3 RESULTS

### 3.1 Distribution of the Basic Vaginal States According to the Contraception Method

The basal set of test results obtained from the 101 women included in the study before they started using the contraceptive method of their choice was as follows: 55 (54.5%) showed BVS I, 16 (15.8%) BVS II, 3 (3%) BVS III, 17 (16.8%) BVS IV and 10 (9.9%) BVS V. Fig. (**1**) shows the distribution and follow-up of the study participants according to the contraceptive method, at the beginning of the study and after three and six months of use.

After three months of contraception, 94/101 women attended and fulfilled the initial study and the evaluation at three months. When the changes in the vaginal microbiota were considered, independently of the variation in VIR, 53 patients maintained BVSs with normal vaginal microbiota (BVS I and II) and 10 of the 25 women with vaginal dysfunction before starting the COCP reverted to normal vaginal microbiota (conversion to BVS I and II). These changes showed a statistically significant association between the COCP and normal vaginal microbiota (p= 0.01) (Table **2**). Of the 7 women who chose the use of condom, 2 evidenced VIR (conversion to BVS II), while the remaining maintained an altered BVS (Table **2**). Of the 8 patients who chose the rhythm method, 7 maintained their initial BVS (Table **2**).

After six months of contraception, 89/101 women attended and fulfilled the initial study and the evaluation at six months. When the changes in the vaginal microbiota were evaluated, independently of the variation in VIR, we found that 50 patients maintained BVSs with normal vaginal microbiota (BVS I and II), and 21 of 24 women with vaginal dysfunction before starting the COCP reverted to normal vaginal microbiota (conversion to BVS I and II). These results showed a statistically significant association between the COCP and normal vaginal microbiota (*p*< 0.0001) (Table **3**). Of the 7 women using condom, 3 showed VIR (conversion to BVS II), which was not present at the beginning of the study (Table **3**). Of the 7 patients who used the rhythm method, 6 retained their initial BVSs (Table **3**).

The distribution of the BVSs in the COCP group after three and six months is shown in Fig. (**2**). An increase is observed in BVS I and II with normal microbiota and a decrease is seen in BVS IV and V with altered microbiota.

### 3.2 Distribution of Yeasts Associated with Basic Vaginal States

In the first evaluation before starting the COCP, yeasts were detected in 25/79 samples. Three months later, the proportion of yeasts was 22/79, but yeasts were found in 7/22 samples that had not presented any yeasts at the beginning of the study (4 with BVS I and 3 with BVS II). It should be noted that only 6 symptomatic women were treated with antifungals (Table **4**).

Before the use of CON, yeasts were detected in only 1/7 sample with BVS II, which remained unchanged after three months. In the RHYT group, at the beginning yeasts were found in 3/8 samples with BVSs I (2) and II (1); after three months, yeasts were observed in one sample with BVS I.

In the first evaluation before starting the COCP, yeasts were detected in 19/75 samples. Six months later, the proportion of yeasts was 12/75, but yeasts were found in 3/12 samples that had not presented any yeasts at the beginning of the study (1 with BVS I and 2 with BVS II) (Table **4**).

Before the use of CON, yeasts were detected in only 1/7 sample with BVS II, which remained unchanged after six months. In the RHYT group, at the beginning yeasts were found in 3/8 samples with BVSs I (2) and II (1); after six months no yeasts were detected.

### 3.3 Distribution of *Trichomonas vaginalis* Associated with Basic Vaginal States

Trichomonas were detected in 11 of the 101 women at the beginning of the study who consulted for the use of COCP and had BVS II (3) and BVS V (8). The 11 patients started treatment (metronidazole 1000 mg daily for seven days) and contraception with COCP. After three months, the parasite was absent in the 8/11 patients attended. After six months, the parasite was absent and the normal vaginal microbiota was restored in the 11 patients (BVS I and BVS II). Trichomonas were not detected in the women who consulted for CON and RHYT.

### 3.4 *Lactobacillus Species * in Vaginal Contents

Eighty-four *Lactobacillus* strains were isolated and identified: 23/84 from 94 vaginal content samples obtained after three months and 61/84 from 89 vaginal content samples obtained after six months of initiating contraception. The genomic and proteomic identification were performed for all the lactobacilli isolates.

The two methods applied showed concordant results in 78 (92.9%) of the isolates. Of these, 27 (34.6%) strains were identified as *L. gasseri*, 25 (32.1%) as *L. crispatus*, 15 (19.2%) as *L. jensenii,* 10 (12.8%) as *L. murinus* and 1 (1.3%) as *L. johnsonii.* The Kappa concordance correlation coefficient obtained between the two methods was 0.901 (Table **5**). Fig. (**3**) shows the different biotypes of the strains obtained during the identification of lactobacilli by rep-PCR assays, that indicates the patterns of all the biotypes, eventhough they were not the dominant species in the study. One representative from each biotype was identified by partial 16S rRNA gene sequencing.

In 7.1% (6/84) of the cases, the results were discordant: 83.3% of these discordances were among related species *L. gasseri* and *L. jensenii*, and 16.7% among *L. gasseri* and *L. crispatus* (Table **5**). The species that were isolated in a higher proportion were *L. gasseri* and *L. crispatus*. The former was isolated from 88.9% of healthy women and 11.1% with bacterial vaginosis (BVS IV). *L. crispatus* was associated with normal vaginal microbiota (100%) and 84% of the strains were isolated from the group of women who chose COCP. *L. murinus* was first isolated from the vagina and in a remarkable proportion.

## 4 DISCUSSION

The uninterrupted use of COCP in both periods (three and six months) did not only maintain the normal microbiota, but also decreased the frequency of bacterial vaginosis. The hormonal contribution of COCP, although it is an “external” factor, represents one of the main regulators of the hormonal action and, therefore, supports the hypothesis of the effect of estrogen on the vaginal state. Similar results were described in a few recent studies, which have only reported the effect of hormones [2].

One of the mechanisms of protection demonstrated, both for endogenous and exogenous sex hormones, is the hormonal effect on the vaginal mucosa through the cell receptors. These receptors stimulate the multiplication of the epithelium and thus help the production of adequate glycogen concentration in intermediate cells. Estrogen stimulation produces a predominance of surface layer cells, whereas progesterone stimulation produces a predominance of intermediate layer cells [21]. In response to hormonal variations, the lysis of the latter cells generates glycogen, thus favoring the survival of lactobacilli and the maintenance of a protective pH [22-24]. In addition, recent studies have documented another hormone-dependent protection mechanism through an activity of proton pumping (by an H+-ATPase) in the apical membrane of vaginal and cervical epithelium cells stimulated by estrogen, which decreases at menopause, with the consequent increase in vaginal pH [25].

However, the results of this research indicate that the use of COCP promoted yeast colonization in healthy women, since the highest proportion was obtained after three months of consumption, with new carriers detected at three months with BVS I and BVS II, in agreement with our group previously studied and with other authors [1-3]. Nevertheless, it cannot be ruled out that this colonization represents a predisposing condition for recurrent vulvovaginitis.

It is well known that physiological and pharmacological hyperestrogenemia is associated with higher yeast colonization in the vagina, which could result from certain mechanisms directly mediated by estrogens, mainly the increase in vaginal glycogen, the reduction of vaginal pH and easier adhesion to epithelial cells. Although this subject has been under study for many years, it remains a controversial issue.

In the *Trichomonas vaginalis* carrier group, after six months, a *Lactobacillus*-dominated vaginal microbiota was recovered with the combined treatment of metronidazole and hormonal contraceptives, in addition to the absence of the parasite. Acting as vaginal sentinels and biomarkers, the predominance of *Lactobacillus* determined the consistency between clinical and microbiological cure rates.

The fact that condoms were used by a low percentage of the women did not allow a comparative analysis with COCP. This study highlights instead that the use of male condoms demonstrated conversion to BVS with VIR (BVS II and BVS V). These results are consistent with previous reports that have shown the presence of vaginal dermatitis, allergic and irritant vulvovaginitis, and inflammation associated with the use of condoms, due to the influence of either the latex or the spermicide [4]. It must be considered that the vulvar mucosa is nonkeratinized epithelia and the moisture in which it is immersed promotes the penetration of irritants and allergens, thus generating vaginitis states [26].

With regard to the rhythm method, the reduced number of women (7/101) adopting this contraceptive method makes it difficult to draw a conclusion or to refer to the results of other researches. However, the balance of the vaginal microbiota turned out to be surprising. The vaginal microenvironment of these women is only under the systemic hormonal influence during the ovulation stage, which favors the growth of lactobacilli and preserves normal microbiota. Also during the sexual abstinence period, there is no exposure to semen or leukocytes as modifiers of the vaginal environment, and therefore it remains stable and normal for longer.

Although with the condom and the rhythm method no significant changes were detected in the proportion of yeasts, due to the small size of both groups, it is necessary to continue the study with a larger number of women to evaluate the influence.

The closely related species within the *L. acidophilus* complex are difficult to differentiate by phenotypic methods, which may account for the variation in *Lactobacillus* species or clusters associated with the vaginal microbiota published by different authors (20). The culture in a specific medium only detects some fastidious organisms and underestimates the diversity of the vaginal microbiome; however, it allows detecting the predominant genus or species, studying the expression of defensive properties and relating them to different conditions of the vaginal microbiota. It is important to identify lactobacilli to evaluate the role of the different *Lactobacillus* species, particularly in relation to their ability to produce H_2_O_2_ and to function as probiotics. We must recognize that our results are limited and biased because certain microbial species, mainly *L. iners,* do not grow easily in laboratory conditions. Under most circumstances, normal vaginal microbiota is dominated by *Lactobacillus* species. Based on the hypothesis of “competitive exclusion” and “bacterial interference” mechanisms, when lactobacilli dominate the vaginal microbiota, culture-independent techniques have shown, and also in this work, that a healthy vagina is usually colonized by only one or two dominant *Lactobacillus* species, mainly *L. crispatus, L. gasseri* and *L. jensenii*. The dominant *Lactobacillus* species may differ racially or geographically, but the principle of numerical dominance continues and could act as a main defense mechanism [13, 27]. The finding of *L. murinus* for the first time in the vagina and in a significant percentage could reflect the relationship between species and geographical locations. In this work, 8 of the 10 carriers of this species are rural workers on dairy farms and consumers of unpasteurized milk, which could explain the detection of this species in the samples.

In this study, MALDI-TOF MS analysis showed 92.9% concordant results with 16S rDNA sequencing applied to identify lactobacilli isolates from vaginal samples at the species level. Consequently, mass spectrometry proved to be an accurate and fast method to identify vaginal lactobacilli species. Other studies comparing mass spectrometry with genotypic methods have reported a greater concordance percentage (96%) [28], a similar concordance percentage (93%) [29] and lower values (74%) [30] in comparison to our results. Most of the discordant results can be explained by the fact that *L. gasseri, L. jensenii* and *L. crispatus* are phylogenetically closely related species. The phylogenetic analysis supports the sequence similarity. The combination of 16S rDNA sequencing and MALDI-TOF MS revealed a new species associated with the vaginal microbiota, *L. murinus*.

The isolation of *L. gasseri* in women with bacterial vaginosis (BVS IV) suggests specific areas for future research, such as assessing the potential role of *L. gasseri* in bacterial vaginosis pathogenesis [31]. Besides, the association between *L. crispatus* and normal microbiota (100%) agrees with the results of Marrazzo *et al.* and is supported by the protective properties of *L. crispatus* to maintain a healthy microenvironment [31].

## CONCLUSION

The pattern of vaginal states was significantly modified according to the type of contraceptive method used. Hormone administration apparently corrected the alterations and preserved a normal vaginal state. MALDI-TOF MS has the potential of being an accurate tool for vaginal lactobacilli species identification in concordance with 16sr-DNA sequencing. *L. murinus* was for the first time isolated from the vagina and may reflect the relationship of the species with geographical locations.

## Figures and Tables

**Fig. (1):**
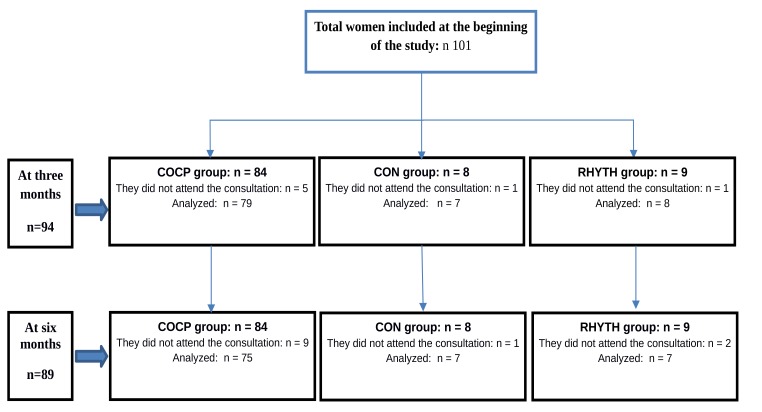
Follow up of the study participants.

**Fig. (2):**
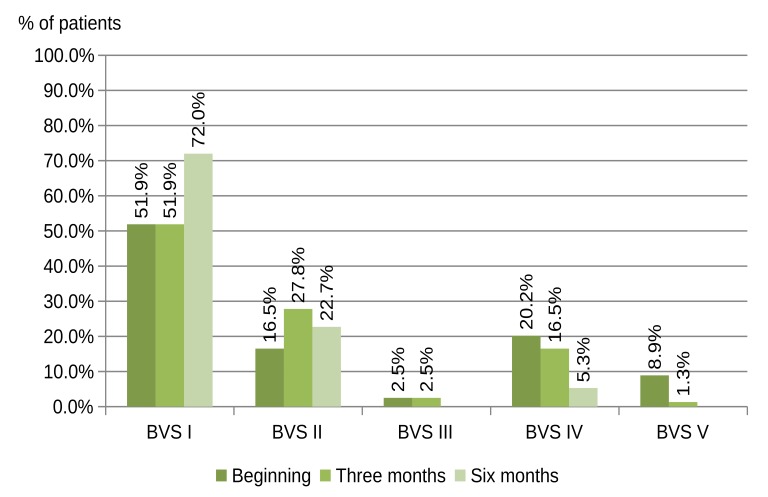
Basic Vaginal States (BVS): Conversion by hormonal contraception.

**Fig. (3):**
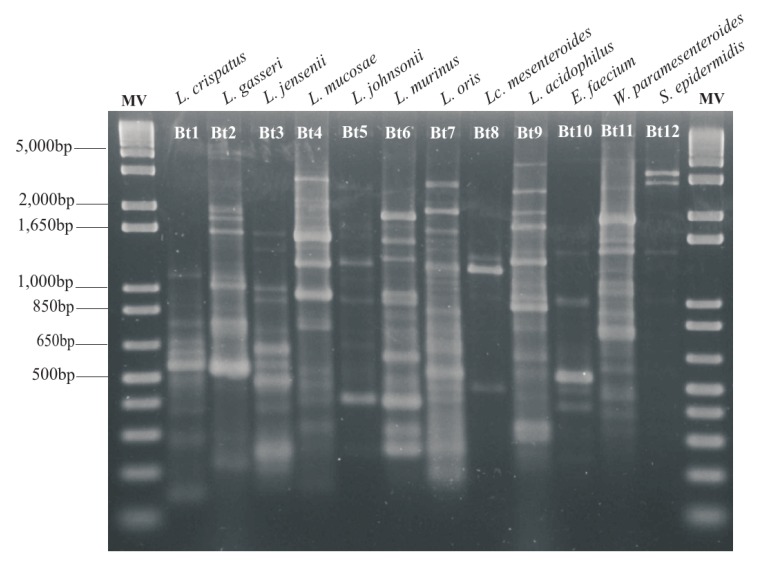
Representative biotypes obtained from Random amplified polymorphic DNA (RAPD)-PCR profiles of lactic acid bacteria (LAB) isolated from vaginal samples. Primers (GTG)5 were used for RAPD-PCR analysis. MW: Molecular weight marker (1kb DNA ladder, Invitrogen). (GTG)5 RAPD profiles including the following biotypes: Bt1 (*L. crispatus,*); Bt2 (*L. gasseri*); Bt3 (*L. jensenii*); Bt4 (*L. mucosae*); Bt5 (*L. johnsonii*); Bt6 (*L. murinus*); Bt7 (*L. oris*); Bt8 (*Lc. mesenteroides*); Bt9 (*L. acidophilus*); Bt10 (*E. faecium*) Bt11 (*W. paramesenteroides*) and Bt12 (*S. epidermidis*). Methods are described in the text.

**Table 1:** Criteria for the definition of the basic vaginal states (BVSs).

**BVSs in Women in Fertile Age **	**NV**	**VIR**
**I **normal microbiota *Predominance of lactobacilli*	0 to 3	No
** II **normal microbiota + vaginal inflammatory reaction *Predominance of lactobacilli, but presence of vaginal inflammatory reaction*	0 to 3	Yes
** III** intermediate microbiota *Balance between lactobacilli and anaerobic bacteria*	4 to 6	No
**IV** bacterial vaginosis *Predominance of anaerobic bacteria*	7 to 10	No
** V **non-specific microbial vaginitis *Alteration of the lactobacilli/anaerobic bacteria relation, with inflammatory reaction*	4 to 10	Yes

BVSs: Basic Vaginal States; NV: Numerical Value (Nugent Criteria); VIR: Vaginal Inflammatory Reaction.

**Table 2:** Evaluation of three months of contraception: Distribution of basic vaginal states* (*BVSs) according to the contraception method.

**BVS^1^**	**COCP^ 2^**		**CON^ 3^**		**RHYT^ 4^**	
	**Initial Study** Distribution of BVS (n)	**After 3 Months** Conversion to BVS Analyzed n= 79	**Initial Study** Distribution of BVS (n)	**After 3 Months ** Conversion to BVS Analyzed n= 7	**Initial Study** Distribution of BVS (n)	**After 3 Months ** Conversion to BVS Analyzed n= 8
I normal microbiota	41	I = 34 II = 7	**3**	I = 2 **II = 1**	**5**	I = 5
**II** normal microbiota +VIR	13 *(2/13)	I = 2 II = 10 III = 1	**2**	II = 2	**2**	I = 1 II = 1
**III** intermediate microbiota	**2**	**I = 1** III = 1	**1**	**II = 1**	**-**	-
**IV** bacterial vaginosis	16 *(1/16)	**I = 3** **II = 2** IV = 11	**-**	-	**1**	IV = 1
**V** non-specific microbial vaginitis	7 *(7/7)	**I = 1** **II = 3** IV = 2 V = 1	**1**	V = 1	**-**	-
**TOTAL**	**79**	**Conversion to BVS I – II of microbiota normal = 10**	**7**	**Conversion** **to BVS II with VIR = 2**	**8**	-

^1^BVSs: Basic Vaginal States - **^2^**COCP: Combined Oral Contraceptive Pill (levonorgestrel-ethinylestradiol) - **^3^**CON: Condom - **^4^**RHYT: Rhythm Method -*Metronidazole Treatment.

**Table 3:** Evaluation of six months of contraception: Distribution of basic vaginal states* (*BVSs) according to the contraception method.

**BVS^1^**	**COCP^ 2^**		**CON^ 3^**		**RHYT^ 4^**	
	**Initial Study** Distribution of BVS (n)	**After 6 Months** Conversion to BVS Analyzed n= 75	**Initial Study** Distribution of BVS (n)	**After 6 Months ** Conversion to BVS Analyzed n= 7	**Initial Study** Distribution of BVS (n)	**After 6 Months ** Conversion to BVS Analyzed n= 7
**I** normal microbiota	**41**	I = 33 II = 7 IV = 1	**4**	I = 2 **II = 2**	**4**	I = 4
**II** normal microbiota +VIR	**10**	I = 6 II = 4	**1**	II = 1	**2**	I = 1 II = 1
**III** intermediate microbiota	**1**	**I = 1**	**1**	**II = 1**	**-**	-
**IV** bacterial vaginosis	**14**	**I = 8** **II = 4** IV = 2	**-**		**1**	IV = 1
**V** non-specific microbial vaginitis	**9**	**I = 6** **II = 2** IV = 1	**1**	V = 1	**-**	-
**TOTAL**	**75**	**Conversion to BVS I – II of microbiota normal = 21**	**7**	**Conversion** **to BVS II with VIR= 3**	**7**	-

**^1^**BVSs: Basic Vaginal States - **^2^**COCP: Combined Oral Contraceptive Pill (levonorgestrel-ethinylestradiol) - **^3^**CON: Condom - **^4^**RHYT: Rhythm Method -*Metronidazole Treatment

**Table 4:** Distribution of yeasts associated with basic vaginal states (BVSs) at 3 and 6 months after the initiation of COCP.

**BVS^1^**	**COCP ^2^**			
	**Yeast Detection** **Analyzed n = 79**		** Yeast Detection** **Analyzed ** **n = 75**	
	*Evaluation of Three Months of Contraception*		*Evaluation o Six Months of Contraception*	
	Initial Study (n)	After 3 Months (**n)**	Initial Study (n)	After 6 Months (n)
**I** normal microbiota	11	8 4/8**	9	5 1/5**
**II** normal microbiota + vaginal inflammatory reaction	9 (4/9)*	13 3/13**	6	7 2/7**
**III** intermediate microbiota	1	-	-	-
**IV** bacterial vaginosis	2	-	2	-
**V** non-specific microbial vaginitis	2 (2/2)*	1	2	-
**total**	**25**	22	**19**	12

^1^BVSs: Basic Vaginal States ^2^COCP: Combined Oral Contraceptive Pill (Levonorgestrel-Ethinylestradiol) *Treated with antifungals **Undetected at the beginning of contraceptive method used

**Table 5:** Degree of agreement in the identification of lactobacilli with MALDI-TOF MS and Rep-PCR genomic fingerprinting.

**Identification Methodology**	**MALDI-TOF MS**					
**Rep-PCR genomic fingerprinting**	***Lactobacillus* species**	***L. crispatus*** **(n)**	***L. gasseri*** **(n)**	***L. jensenii*** **(n)**	***L. johnsonii*** **(n)**	***L. murinus*** **(n)**
	*L. crispatus* (n)	**25**	-	-	-	-
	*L. gasseri* (n)	1	**27**	5	-	-
	*L. jensenii* (n)	-	-	**15**	-	-
	*L. johnsonii* (n)	-	-	-	**1**	-
	*L. murinus* *(n)*	-	-	-	-	**10**
***Kappa Coefficient: 0.901****(excellent degree of concordance)* *Confidence interval Kappa SE(0): (0.775, 1.028)* * Confidence interval Kappa SE(1): (0.826, 0.977)*						

## LIST OF ABBREVIATIONS

**COCP**: = Combined Oral Contraceptive Pill
CON: = Condom
RHYT: = Rhythm Method
VIR: = Vaginal Inflammatory Reaction
**BVSs**: = Basic Vaginal States
